# Development of a curriculum and roadside screening tool for Law enforcement identification of medical impairment in aging drivers

**DOI:** 10.1186/s40621-016-0078-3

**Published:** 2016-05-02

**Authors:** Linda L. Hill, Jill Rybar, James Stowe, Jana Jahns

**Affiliations:** 1 University of California, 9500 Gilman Drive #0811, La Jolla, San Diego, CA 92093-0811 USA; 2 Washington University in St. Louis, School of Medicine, 660 S. Euclid Ave., Campus Box 8303, St. Louis, MO 63110 USA; 3 Missouri Coalition for Roadway Safety, Subcommittee on Elder Mobility and Safety (SEMS), Jefferson City, USA

**Keywords:** Roadside screening tool, Medically impaired driver, Orientation, Motor vehicle crash (MVC)

## Abstract

**Background:**

An estimated one in five drivers will be over 65 by 2030. Compared with their younger counterparts, older adults are more likely to experience health and functional impairments, including cognitive dysfunction, which may interfere with their ability to drive safely. Law enforcement officers, as part of the public safety community, need help in developing the necessary skills to identify and manage these medically affected drivers.

**Methods:**

To address this need, in partnership with the California Highway Patrol (CHP), Training, Research and Education for Driving Safety (TREDS) at the University of California, San Diego, developed a certified two-hour training curriculum. To complement the training, the TREDS team also developed a roadside screening tool to assess for disorientation related to person, place, and time. The tool was developed, validated with a sample of persons with dementia compared to cognitively normal controls, and deployed in the training. A total of 2,018 police officers received instruction at 103 training sessions.

**Results:**

At baseline, prior to training, only 26 % of officers had reported drivers to the Department of Motor Vehicles in the previous 6 months. After training, 96 % stated they were likely to use their standard reporting forms, and 90 % reported they were likely to use the roadside screening tool.

****Conclusion**s:**

The certified training and tool were well received and resulted in changes to knowledge, attitudes, and intention to incorporate their new knowledge and tools into roadside screening.

## Background

Medical conditions often cause functional impairments that can affect driving performance, and medically impaired drivers are overrepresented in crashes. By 2030, one in five drivers will be over 65 (Ortman et al. 2014). As drivers enter advanced age, many are at greater risk of experiencing reduced visual, cognitive, or psychomotor function, including impairments caused by the side effects of medication. While many older adults self-regulate and retire from driving when they are no longer able to safely operate a motor vehicle, a significant number do not (Wong et al. 2015). Fatal crash involvement per 100 million miles driven for adults over 80 is the highest of all age groups, including teens, however due to reduced miles driven, crash rates per age-specific population are much lower (McCartt & Teoh 2014).

Even in health care settings, medical impairment is subtle, making it difficult to identify and diagnose. Unless the physician has been trained to recognize the symptoms and systematically screen for them, the condition may escape notice (Chodosh et al. 2004). Reinforcing this tendency, they remain less prone to self-regulate their driving than their healthy counterparts (Wong et al. 2012), though certain types of feedback may help promote self-regulation in impaired participants (Ackerman et al. 2014). In addition, physicians can be reluctant to broach the topic of medical fitness to drive because they have not been adequately trained on fitness-to-drive screening or discussions, and may worry about alienating their patients (Betz et al. 2013).

At the same time, law enforcement officers report leniency with this group and have sometimes failed to alert licensing agencies about medically impaired drivers until *after* a crash or other traffic incident (Meuser et al. 2009). Therefore, for law enforcement and medical professionals, the problem lies both in *identifying* medically impaired drivers and *responding* appropriately to their conditions. The focus of this study is on law enforcement.

Compounding the lack of training and response across professional domains related to medical impairment and driving is the increase in the prevalence of dementia in aging drivers (Carr et al. 2006). Some law enforcement officials use the term “low-volume, high-risk” for these types of drivers (Hill et al. 2014), and analyses of state reports of medically impaired drivers suggest dire consequences from failure to remove these drivers from the roadway (Meuser et al. 2009). Even if encounters with medically impaired drivers continue to be relatively rare, appropriate training of and response by law enforcement will reduce adverse outcomes.

In recognition of the effect of medical impairment on driving, the National Highway Transportation Safety Administration (NHTSA) developed the *Older Driver Program Five-Year Strategic Plan 2012-2017* that addresses the “need [for law enforcement to have] more training and information to help them better assess warning signs and understand what next steps to take and what actions are mandated by laws within their jurisdiction” (NHTSA 2010, p.10). This need is also documented in *California’s Strategic Highway Safety Plan to Improve Safety of Older Roadway Users (*http://www.dot.ca.gov/hq/traffops/shsp/docs/SHSP15_Update.pdf) with this recommendation: “Enhance law enforcement training to recognize older driver behaviors that may necessitate priority drivers’ license re-examinations, and provide law enforcement with a broader understanding of older driver sensitivities.”

Along with California, many other states are considering and implementing countermeasures to prevent the involvement of medically impaired older drivers in collisions that cause serious injuries and fatalities. In part, these countermeasures have been motivated by language in *Moving Ahead for Progress in the 21st Century* (MAP-21), a surface transportation bill that recommended the inclusion of strategies for older adults in the state’s *Strategic Highway Safety Plan* (SHSP) if the state experienced an increase in older driver or pedestrian injuries and fatalities.

These signals point to the need to develop effective training materials and roadside screening tools that help law enforcement officers identify signs of medical impairment and give them appropriate protocols to respond. Moreover, the value of such training and tools must be reinforced with law enforcement administrators so that they encourage their officers to undergo the training and make use of the tools in their day-to-day roadside stops.

### Existing training does not lead to identification of medical impairment

In many jurisdictions, law enforcement plays a crucial role in screening potentially impaired drivers who may benefit from further evaluation of medical fitness-to-drive by physicians or professional driver examiners. Two recent analysis from Maryland (Soderstrom et al. 2009; Soderstrom et al. 2010), echoed by analyses from Virginia (Lococo et al. 2013) and Missouri (Meuser et al. 2009) suggest that, while law enforcement reports that recommend medically impaired drivers to licensing authorities for further evaluation of medical fitness-to-drive are quite effective, the *volume* of such reports remains quite small. Because these reports play such a crucial role in public safety, the impact of training on increasing their quantity and efficacy should be investigated more broadly.

### Need for improved training and screening tools

Screening tools for medical issues related to cognitive impairment exist (e.g., Trailmaking Tests A and B, Clock Drawing Task, etc.; see Fonseca et al. 2015; Nair et al. 2010), but they may be impractical for roadside administration due to materials commonly necessary for these tasks (e.g., paper and pencil; timer) and/or relatively complex scoring criteria. Moreover, most existing tools fail to provide law enforcement professionals appropriate guidance to cite and report impaired drivers to relevant licensing authorities. For a tool to be effective and not just useful but *used* in roadside encounters, it must be brief, easy to administer and score (e.g., not require involved test sequences or scoring protocols), and support a definitive decision to guide the officer’s next actions.

To address this need, Training, Research and Education for Driving Safety (TREDS) at the University of California, San Diego, developed a curriculum to train law enforcement officers to identify medically impaired drivers. With support from the California Office of Traffic Safety (OTS), TREDS partnered with the California Highway Patrol (CHP) and the California Department of Motor Vehicles (DMV) to develop a curriculum to train law enforcement officers in the CHP Border Division, which encompasses four counties in southern California. The curriculum, based on NHTSA’s *Law Enforcement Training on Older Drivers,* was first released in 2007, followed by several major course revisions, including content tailored specifically to state law and officers’ needs. To complement this training, TREDS also developed and validated a roadside screening tool to assess possible driver medical impairment. This tool is called the Driver Orientation Screen for Cognitive Impairment (DOSCI). To our knowledge, no other tool to screen potentially disorientated drivers has been developed and validated for application during roadside stops.

We employed a qualitative approach for initial training and instrument design (e.g., focus groups and expert interviews), followed by pre- and post-training questionnaires to assess the efficacy of the training and initial validity of the screening tool. During development and initial validation, the following research questions were addressed:

- What are the nature and content of training that will enhance law enforcement’s ability to identify and respond appropriately to disoriented drivers?
- Is the DOSCI a valid, practical tool for field use by law enforcement to identify, manage, and appropriately report disoriented drivers?

## Methods

Initial training and tool development started in 2010. The training sessions included in the analysis (Table 1) were conducted from 2011 through early 2015. Activities reported in this study span initial training and screening tool development to long-term follow up on training and tool utility and efficacy.

**Table 1:** Training sessions and attendance

	2010–11	2011–12	2012–13	2013–14	2014–15	TOTAL
# of trainings	41	26	30	5	1	103
# of officers	699	561	664	75	19	2,018

### Participants

Four groups of participants were used for this training and screening tool development study:

- Group 1: Experts and a focus group (*n* = 12) were used to identify initial content and potential design of the screening tool. This group was recruited from local police and highway patrol, identified by their peers as knowledgeable and representative of their group.
- Group 2: After creation, the DOSCI tool was initially validated using 68 patients from the Alzheimer’s Disease Research Center. These participants were identified by the ADRC as presenting to the center for other testing during the 6 months of data collection in 2012. The ADRC identified persons with and without the diagnosis of dementia during this period for inclusion.
- Group 3: Evaluation of the training curriculum’s efficacy involved data from 103 sessions that trained law enforcement officers from state and local agencies (*n* = 2,018) over a five-year period. TREDS staff and consultants delivered 96 classes to the CHP training 1,946 officers. Seven classes were delivered to local police agencies training 72 officers. This group included officers in the Border Division and San Diego County who were required by their group to attend trainings, and were representative of other trainees.
- Group 4: Long-term follow up with state law enforcement officers occurred 24 months post receipt of the training curriculum (*n* = 60). These individuals were known to have taken the training, and were interviewed during shift changes.

### Training curriculum development

Initial content for the training and screening tool was developed based on interviews with law enforcement leaders and a subsequent focus group. The TREDS team used basic content analysis to categorize the data into meaningful themes (Hsieh & Shannon, 2005).

Interview respondents were queried about state-specific older-driver issues (statistics, reporting, current programs); their experiences with the NHTSA curriculum, teaching strategies, and curriculum content; and approaches for incorporating the training into their in-service training programs. Additional issues specific to California law were included, such as mandated reporting by physicians and law enforcement reporting to the DMV.

At the recommendation of these experts, we conducted a focus group with 12 officers in the CHP Border Division. This two-hour session addressed the types of training likely to be successful, teaching strategies for officers, and local issues. We also gathered feedback on a variety of mechanisms to determine possible driver disorientation during a traffic stop.

A skilled focus group facilitator was given a semi-structured guide, and provided information on various training options that could be used, and participants were asked to provide feedback on their preferences. The facilitator helped structure the feedback, and all participants had the opportunity to voice their opinions. Training options included format of the information, use of videos, role playing, question and answer, the number of presenters, the backgrounds of presenters, and the length of the presentation. These options were framed in light of the officers’ perceived available time, their baseline knowledge of the issue of medically impaired drivers, and their desired training outcomes. The focus group session was based on social cognitive theory, also serving as the basis for the training curriculum.

The expert interviews and focus group provided guidelines with which to structure the training curriculum, including the following:

- Consensus of two hour training length
- Presentation methods (frequent changes of format and use of videos, slide shows, and rotating speakers were preferred)
- Inclusion of a law officer presenter with traffic experience
- Case scenarios

The expert interviews and focus group together helped effectively translate research knowledge on medical driver impairment to the specific context of law enforcement’s use of the training and screening tool in the field.

The resultant two-hour curriculum (Table 2) was titled “Older Driver Safety: Law Enforcement’s Role.”

**Table 2:** Training curriculum outline

Topic	Learning Objective Officer Will Understand	Personnel
Aging and Medical Conditions	How age-related changes in health and functional status impair driving ability and increase crash risk	Physician
Older Driver Traffic Stop	Approaches to identifying impairment in older drivers	Law Enforcement
Driver Re-examination	Enforcement actions for documenting suspected impairment	Department of Motor Vehicles
Driver Education and Evaluation	Resources to assist aging drivers maintain mobility	Health Educator

**Table 3:** Pre- and post-training questionnaires completed

Category	2010–11	2011–12	2012–13	2013–14	2014–15	Total
# of pre-training questionnaires completed	651	530	634	74	16	1,905
# of post-training questionnaires completed	681	550	661	74	19	1,985

The training covered the following topics:

- Introduction to training and older drivers
- Medical conditions and methods for assessment (addressing issues of vision, frailty, cognitive impairment, and hypo- and hyperglycemia)
- Strategies to employ during traffic stops (including observation, questioning, use of the screening tool [described below], and communication and referral)
- Use of the DMV reporting mechanism
- Community resources for driver evaluation and re-education

Local and state law enforcement professionals both received training. Data from the two groups were combined for analyses and reporting.

### Development of the DOSCI roadside screening tool

**Fig. 1:**
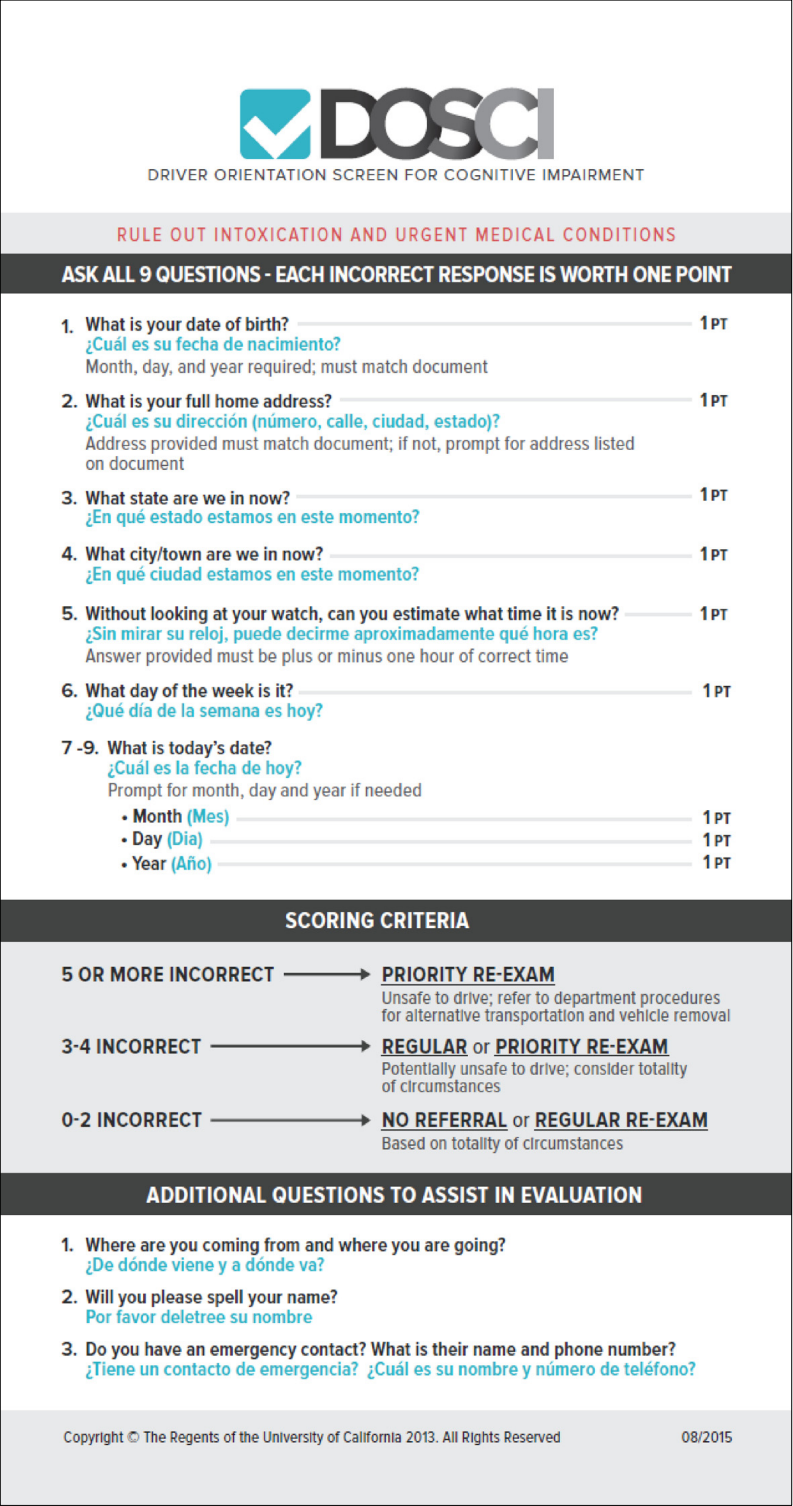
DOSCI roadside screening tool

To complement the training officers received and help them implement it in their day-to-day traffic work, the TREDS group developed an easy-to-use screening tool for disorientation (Fig. 1), called the DOSCI. The tool was to be used to help clarify whether the confused and possibly disheveled patient was disoriented, and to provide a reminder and guidance for reporting medically impaired drivers. The expert interviews and focus group (described above) produced recommendations that the DOSCI:

- Be short and simple to use
- Include questions that assess for orientation to person, place, and time
- Avoid other methods to assess memory such as drawing and counting exercises

In California, highway patrol officers are trained on roadside testing for alcohol and drug impairment; they have the ability to call in Drug Recognition Experts. Once intoxication from alcohol, impairment from prescription medication or illicit drugs, and medical conditions requiring urgent care have been ruled out, the officer administers the DOSCI to determine possible disorientation. It was designed to fit conveniently in an officer’s ticket book. It lists nine items (in English and Spanish) to ask a person stopped for a traffic violation who presents signs of possible disorientation (e.g., who appears confused or is slow to answer questions). It includes scoring guidelines and reporting recommendations for further medical evaluation of fitness-to-drive by the state licensing authority. Scores that are weighted equally for each item or item component are then summed, and the total score is trichotomized for unlikely, potential, and likely disorientation.

The DOSCI was validated at the UCSD Alzheimer’s Disease Research Center. It was administered to 41 patients with dementia (13 female, average age 74) and 27 cognitively intact control patients (12 female, average age 77) during regularly scheduled visits during which patients return to the center approximately every six months for measurement and evaluation.

### Collection of questionnaire data

Pre- and post-training questionnaires were developed and pilot-tested to measure the impact of the training. Descriptive statistics were used to characterize differences in the answers between pre- and post-training among officers trained using the curriculum.

Pre-training questions addressed basic demographics, confidence in identifying disorientation in older drivers, experiences with older drivers, assessment practices, DMV reporting practices, and referrals of potentially impaired drivers for evaluation of medical fitness to drive. Post-training questions addressed understanding of the curriculum, perceived changes in confidence, intention to assess and report when indicated, and intention to use the screening tool.

Filling out the pre- and post-training questionnaires was anonymous and voluntary, and included an open-ended section for comments. Questionnaires were completed in a classroom setting, immediately preceding and following the training. Overall responses were received for 1,905 (96.6 %) pre-tests and 1,985 (98.4 %) post-tests. All officers were given pre and post tests, though not all completed them (Table 3).

### Data analysis

The tool validation study was assessed used unpaired *t*-tests for group comparison and statistical significance was set at *p* < .05. Differences pre- and post-training were assessed using frequency analysis of self-reported screening and reporting of potentially impaired drivers, and qualitative analysis of participant comments. Post-training semi-structured interviews were qualitatively analyzed to offer insight into feasibility and acceptability of the training and screening tool when used in the field.

### Long-term follow up

Two years after receiving the training, 60 CHP officers in the Border Division participated in interviews to assess training retention and current use of the DOSCI tool. Analyses of the follow up was completed using descriptive statistics from semi-structured, in-person, one-on-one interviews that included survey responses and content analysis of qualitative responses to open-ended items.

## Results

### DOSCI validation study

**Fig. 2:**
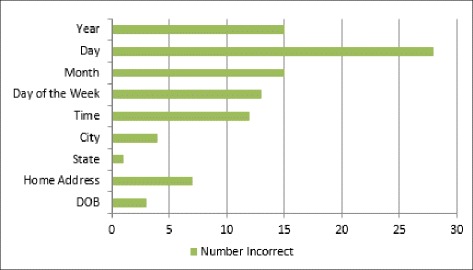
DOSCI tool validation: Incorrect responses by subjects with dementia

To validate the DOSCI, independent samples *t-*tests were used to assess the significance of differences in mean scores between individuals with dementia from the Alzheimer’s Disease Research Center and cognitively normal controls. Mean numbers of errors on the nine-item DOSCI were compared. Independent unpaired *t-*tests indicated that the mean number of errors was 2.36 (SD 2.14) incorrect answers for patients with dementia, compared with only 0.22 (SD .51) (*p* < .0001) incorrect answers for control patients (Df = 66). Day, year, address, and day of the week were the most commonly missed items, while state and date of birth were least commonly missed (Fig. 2).

Based on the ADRC testing, suggestions on driving options and reporting options were included the pocket card (and to remind officers of reporting options to the DMV). Our law enforcement and medical consultants, based on their experience and the ADRC testing, provided guidance on the included advice as follows:

- Five or more incorrect answers (out of nine answers/points total) suggest the person is unsafe to drive that day, and the officer should refer to departmental procedures for alternative transportation and vehicle removal.
- Three to four incorrect answers suggest the person is *potentially* unsafe to drive that day and, based on other circumstantial indications, might be referred for a re-examination at the DMV or immediately restricted in the ability to drive.
- One or two incorrect answers suggest no referral is needed based on the results of the orientation assessment alone.

If an officer needs more information to make a decision confidently, s/he can ask the following supporting questions from the DOSCI instrument:

- Where are you coming from, and where are you going?
- Will you please spell your name?
- Do you have an emergency contact? What is that person’s name and phone number?

This information may help to clarify the presence of confusion and disorientation. The officer can also listen for telling comments the driver might make that indicate problems with driving, previously getting lost, or general disorientation.

Of the 2,018 officers trained (Table 1), 1,905 (94 %) completed pre-training questionnaires and 1,985 (98 %) completed post-training questionnaires. Of the trained officers, 72 were local law enforcement; 72 (100 %) completed pre-training questionnaires and 70 (97 %) completed post-training questionnaires. The variance between the number of officers trained and the number of questionnaires completed is primarily due to officers arriving to the training late or departing early. However, a small percentage of participants may have elected not to complete the questionnaires.

In the pre-training questionnaires, officers reported low levels of driver referral to the DMV, with only 39 (3.5 %) reporting at least once a month, 259 (23 %) once every three-six months, 599 (53.5 %) once a year or every few years, and 223 (20 %) never having reported.

Law enforcement responded favorably to the training. In the post-training questionnaires, participants stated they intended to be more thorough when evaluating seemingly impaired older drivers: 1,898 (96 %) were likely or very likely to use the state’s form to report a driver suspected of medical impairment to the licensing authority (Notice of Priority Re-examination of Driver [DS 427]), and 1,871 (95 %) agreed or strongly agreed they had a better understanding of the form and reporting process.

In both pre- and post-training questionnaires, officers were surveyed on their ability to recognize disorientation and other medical impairments in drivers. Interestingly, in the pre-training questionnaire, 1,267 (67 %) agreed or strongly agreed that they were confident in their ability. However, in the post-training questionnaire, 1,455 (74 %) stated their ability to recognize medical impairment had increased by at least 50 %. Overall, 1,858 (94 %) reported that the training was useful and effective.

Officers also reported they were inclined to use the DOSCI: 1,770 (89.5 %) were likely or very likely to use the screening tool to assess drivers for disorientation. In addition, 1,828 (93 %) of officers thought that the DOSCI would help in preparing the state reporting form for possible medical impairment and request of re-examination. Importantly, 1,846 (93 %) also reported a better understanding of community resources available to older drivers.

### Results from follow-up interviews

In the follow-up interviews two years after training, 90 % (*n* = 54) of officers reported routinely carrying the state reporting form in their patrol vehicles, and 67 % (*n* = 40) indicated that they still had the DOSCI card in their possession. 23 % (*n* = 14) indicated they had used the DOSCI tool to evaluate an older driver for disorientation, and all respondents indicated that the tool was practical and easy to use. Nearly all respondents (*n* = 58) indicated that the training had better prepared them to handle encounters with older drivers.

## Discussion

### Importance of the topic

Officers provided considerable positive feedback on the importance of this topic, and many expressed enthusiasm at having received the training. As a group, they confirmed that their past reluctance to address this issue during roadside stops had been due to their “respect for elders” and concern about missing “more important violators” due to more time being spent with older drivers. By the training’s conclusion, officers recognized the importance of their roles in protecting older drivers (and others such drivers might adversely affect) from preventable tragedy.

The curriculum developed was deemed usable and effective, and resulted in changes to knowledge, attitudes, and, based on the two-year follow up, behavior. The subject matter fit well with other officer training programs and augmented existing practice and protocols. The mixed-media, multi-speaker format of the training maintained officers’ attention, and inclusion of an interdisciplinary team of presenters provided credibility with respect to the breadth of the material covered. The majority of veteran officers, those with more than 15 years of experience, rated the training highly.

The DOSCI tool was also well received and found to be usable and useful in the field. Officers’ reports of enhanced ability to identify driver disorientation were not surprising as problems with orientation can easily go unnoticed, especially if conversation or questioning is minimal and officers have not received appropriate training targeted to this community. The tool was intended to support the officers in assessing the confused or inappropriate driver.

### Importance of clarifying the reporting process

Including the DMV in the curriculum was important to those officers who previously lacked clarity on reporting. (Often, reporting procedures are not well documented or advertised by licensing authorities because of a lack of a budget for outreach activity.) Of particular importance was how to differentiate between regular and priority requests for re-examination (Soderstrom et al. 2009; 2010). Clarifying this process is critical to enable law enforcement officers to identify and respond effectively to impaired drivers.

In states like Missouri, 30 % of reporting of medically impaired drivers is by law enforcement, and the drivers identified and reported were among the highest risk for crashes (Meuser et al. 2009). These data indicate that appropriate use of established reporting pathways by law enforcement is important to crash prevention.

### POST accreditation crucial to acceptance and use

Tools and training must be translated appropriately to field use, especially within the unique work environment of law enforcement. This training curriculum has received certification from the California Commission on Peace Officer Standards and Training (POST). POST accreditation was crucial to acceptance of the curriculum by the officers and their supervisors, providing professional validation and a mechanism for the training to be incorporated into standard in-service training courses. It has encouraged more officers to take the training.

### Post-implementation addition of traffic stop video

After one year of training and based on officer feedback, TREDS developed an eight-minute video modeling a traffic stop with an older driver and use of the DOSCI. The video was made with the participation of a CHP officer and a local actor based on a script developed by law enforcement consultants and TREDS. The video was then incorporated into the two-hour training. This video was viewed as an important addendum to the training, as high-impact, short-duration materials are well suited to time-constrained professionals.

### Strength and limitations of this study

The strength of this study is based on many factors including support of the development and implementation of orientation screening training by a key law enforcement stakeholder. This type of agency buy-in is critical for field implementation of law enforcement aids. Moreover, a multidisciplinary team ensured the development of a robust training curriculum and screening tool that is sensitive to the needs of the targeted user group. Finally, feedback was received from a high percentage of participants.

This study, however, included mainly CHP officers, which may limit its generalizability. Moreover, state law enforcement professionals generally focus on traffic safety and may have fewer competing priorities than other types of law enforcement professionals. However, the sample of local officers tested showed no significant differences with the state officers, suggesting that the curriculum and screening tool are well positioned for additional testing of external validity in more varied geographic and jurisdictional settings. This training will also be useful for younger populations with medical impairments, including conditions associated with disorientation.

The limitations of this study include the small sample size of local police officers (though we are in the process of increasing the size of this group) and the small validation sample size. However, there were significant differences between the cognitively impaired group and the normal controls, suggesting initial validity of the construct. Due to confidentiality of both officers and the drivers stopped in the course of duty, there is a lack of data on the outcomes of reported drivers by trained officers. At this time, there is a project underway to capture that data.

### Future research

TREDS is currently involved in developing a train-the-trainer model with the CHP to expand curriculum delivery throughout California and in 30-minute “shift-change” trainings for local law enforcement. The DOSCI is being used in other states by officers and DMVs. For example, the Missouri Coalition for Roadway Safety’s Subcommittee on Elder Mobility and Safety (SEMS) recognized the importance of the DOSCI tool and has adapted it to meet Missouri’s reporting requirements. The impacts of these efforts will be evaluated and reported in future studies.

To improve the ease of use of the DOSCI and facilitate collection of anonymous data on the frequency and outcomes of its use, we have developed and plan to pilot-test a smartphone web application. It will be easily alterable to map to an individual state or other jurisdiction’s protocol for how an officer, subsequent to identifying a possibly impaired driver, should proceed. This application will undergo feasibility testing in California and Missouri. To our knowledge, no other screening tool of potentially medically impaired drivers has been developed and validated for application during roadside stops.

## Conclusions

This collaborative study involving health and law enforcement professionals addressed the important public safety/public health issue of how to identify and manage disorientation and other medical impairments in drivers. It translated science-based data into a practical training curriculum and roadside screening tool that found initial implementation success in the field during traffic stops. The two-hour, multidisciplinary, multimedia curriculum addressing medical conditions in older drivers was well received and resulted in positive changes to officers’ confidence in identifying and intent to manage these encounters. As a result of the training, officers were likely to use the DOSCI roadside screening tool and to report potentially impaired drivers. The tool was validated in the field, found to be useful, and has the potential for application at DMVs and similar venues. Future studies will assess the generalizability of the training, the outcomes of the train-the-trainer model, and the effectiveness of the DOSCI web application.

As the U.S. population continues to age, individuals with medical conditions that cause functional impairments behind the wheel will be disproportionately represented in traffic collisions. In this context, enhanced training and easy-to-administer supporting tools are needed by law enforcement. Using the type of structured, evidence-based approach described here will help increase the safety of medically impaired drivers and limit their negative impact on the general driving public. This approach may also serve to identify opportunities for enhanced and new community support and services for these high-risk individuals.

## Abbreviations

CHP: California Highway Patrol
DMV: Department of Motor Vehicles
DOSCI: Driver Orientation Screen for Cognitive Impairment
MAP-21: *Moving Ahead for Progress in the 21st Century* (report)
NHTSA: National Highway Transportation Safety Administration
OTS: (California) Office of Traffic Safety
POST: (California Commission on) Peace Officer Standards and Training
SEMS: (Missouri Coalition for Roadway Safety’s) Subcommittee on Elder Mobility and Safety
SHSP: *Strategic Highway Safety Plan* (report)
TREDS: Training, Research and Education for Driving Safety (the research group at the University of California, San Diego, that conducted this study)
